# Immune responses against *Helicobacter pylori*-specific antigens differentiate relapsing remitting from secondary progressive multiple sclerosis

**DOI:** 10.1038/s41598-017-07801-9

**Published:** 2017-08-11

**Authors:** Georgios Efthymiou, Efthymios Dardiotis, Christos Liaskos, Emmanouela Marou, Vana Tsimourtou, Eirini I. Rigopoulou, Thomas Scheper, Alexandros Daponte, Wolfgang Meyer, Lazaros I. Sakkas, Georgios Hadjigeorgiou, Dimitrios P. Bogdanos

**Affiliations:** 1 Department of Rheumatology and Clinical Immunology, University General Hospital of Larissa, Faculty of Medicine, School of Health Sciences, University of Thessaly, Viopolis, 40500 Larissa, Greece; 2 Department of Neurology, University General Hospital of Larissa, Faculty of Medicine, School of Health Sciences, University of Thessaly, 40500 Larissa, Greece; 3 Cellular Immunotherapy & Molecular Immunodiagnostics, Biomedical Section, Centre for Research and Technology-Hellas (CERTH) - Institute for Research and Technology-Thessaly (IRETETH), 41222 Larissa, Greece; 4 Department of Internal Medicine, University General Hospital of Larissa, Faculty of Medicine, School of Health Sciences, University of Thessaly, 40500 Larissa, Greece; 5 Institute of Experimental Immunology, affiliated to EUROIMMUN AG, Lubeck, Germany; 6 Department of Obstetrics and Gynecology, University General Hospital of Larissa, Faculty of Medicine, School of Health Sciences, University of Thessaly, Viopolis, 40500 Larissa, Greece

## Abstract

To assess whether *Helicobacter pylori* (Hp) antibody (ab) reactivity against individual Hp antigens is pathogenetically relevant to multiple sclerosis (MS), we systematically investigated prevalence and clinical significance of abs against 14 immunodominant and subdominant Hp antigens by ELISA and immunoblotting in 139 consecutive MS patients with relapsing-remitting (RRMS, n = 102) or secondary progressive (SPMS, n = 37). Sera from 39 patients with Parkinson’s disease (PD), 21 with Alzheimer’s disease (ALZ) and 68 healthy controls (HCs), were also tested. Anti-flagellin (18.3%) and anti-p41 (25.0%) abs in MS were less frequent than in HCs (39.4%, 48.5%, respectively). Abs against 5 of the 14 antigens were less frequent in RRMS than HCs, including p41, p54-flagellin, p29-UreA, p67-FSH, and p120-CagA. Anti-VacA abs were more frequent in SPMS than in HCs (42.1 *vs* 12.1%, *p* = 0.019). Anti-p54, anti-p29-UreA and anti-p26 correlated with extended disability status scale (EDSS) (*p = *0.017, *p = *0.005, *p = *0.002, respectively). Anti-p26 and anti-p17 correlated with the number of relapses (*p = *0.037 and *p = *0.047, respectively). This is the first comprehensive analysis of ab reactivities against most Hp antigens in MS patients. Ab responses differ between MS and HCs and between RRMS and SPMS, being more prevalent in SPMS than RRMS, thus suggesting an association between anti-Hp and the former type of MS.

## Introduction

Several studies in the past have suggested that *Helicobacter pylori* (Hp), a common bacterium which causes gastric ulcer, is a potential trigger of multiple sclerosis (MS), mainly due to studies demonstrating an increased prevalence and higher titres of anti-Hp antibodies in patients with MS^1–4^. More recent reports, however, have claimed a protective role for Hp in MS, based on the lower prevalence of anti-Hp antibodies in large series of MS^5–10^. Some investigators have gone a step further to propose specific mechanisms responsible for the protective effect of Hp based on the influence of individual antibody reactivities to Hp proteins in the immunobiology of MS^11^.

We and others have focused on the responses against highly-immunogenic proteins, such as those against heat shock proteins (hsp), as microbial anti-hsp reactivities have been considered potential triggers of autoimmune diseases^12–16^. However, we recently demonstrated lack of specificity of anti-hsp60 Hp antibody responses in MS^14^,, and our data suggested that the likelihood of hsp60 Hp involvement in the microbial-induced pathogenesis of MS is rather negligible. Therefore, Hp antigens other than hsps may play a role in breaking tolerance to MS antigens^14^.

In this study we followed a more systematic approach and tested by immunoblotting antibody reactivity to 14 most immunogenic Hp antigens, including the major Hp antigens currently used for the diagnosis of infection. Some of these are also important for the infectivity of the bacterium. As differences in the type of MS may have an impact on *H*. *pylori* status or *vice versa*, we tested reactivity to Hp antigens in a large number of relapsing-remitting MS (RRMS) and secondary progressive MS (SPMS).

To our knowledge, this is the most comprehensive analysis conducted so far on antigen-specific anti-Hp antibody reactivity in RRMS and SPMS. We found that specific Hp antigens are differentially recognized by MS compared to healthy control (HC) individuals. Antibody reactivity to certain Hp antigens differs in frequency and magnitude between RRMS and SPMS, and is associated with specific clinical features.

## Results

### IgG anti-Hp antibodies in patients with MS

IgG anti-Hp antibodies were assessed by ELISA (Table 1). Amongst the 139 MS patients, 60 (43.2%) were anti-Hp positive (anti-Hp(+)), including 41(40.2%) RRMS and 19(51.4%) SPMS patients (*p* = ns). Anti-Hp antibodies were present in 33 (48.5%) HCs, (MS *vs* HC: *p* = ns, RRMS *vs* HC, p = ns; SPMS *vs* HC, p = ns, RRMS *vs* SPMS, *p* = ns), 14 (35.9%) PD (MS *vs* PD: *p* = ns) and 10 (47.6%) ALZ (MS *vs* ALZ: *p* = ns). Since anti-Hp antibody seropositivity increases with age^17^, MS patients and healthy controls were stratified according to age into two groups (<40 and ≥40 years). Anti-Hp antibodies were present in 23/62 (37.1%) MS patients of ≤40 years compared to 37/77 (48.1%, *p* = ns) of >40 years; anti-Hp antibodies were present in 3/15 (20%) HCs of ≤40 years compared to 30/53 (56.6%, *p* = 0.018) of >40 years. There were not many PD and ALZ patients <40 years to be analyzed.

**Table 1:** Major demographic and clinical characteristics of 139 patients with multiple sclerosis (MS), including 102 with relapsing remitting MS (RRMS) and 37 with secondary progressive MS (SPMS); 68 healthy controls (HC), 39 patients with Parkinson’s disease (PD) and 21 patients with Alzheimer’s disease (ALZ) were also tested.

Characteristics	MS (n = 139)	RRMS (n = 102)	SPMS (n = 37)	HC (n = 68)	PD (n = 39)	ALZ (n = 21)	*p* (MS vs HC)	*p* (RRMS vs SPMS)	*p* (RRMS vs HC)	*p* (SPMS vs HC)	*p* (MS vs PD)	*p* (MS vs ALZ)
Sex (M/F)	41(29.5%)/98(70.5%)	32(31.4%)/70(68.6%)	9(24.3%)/28(75.7%)	28(41.2%)/40(58.8%)	19 (48.7%)/20(51.3%)	8 (38.1%)/13 (61.9%)	ns	ns	ns	ns (0.093^†^)	**0.034** ^**†**^	ns
Age	43.2 ± 12	39.2 ± 9.8	54.5 ± 10	47.4 ± 16.9	68.7 ± 9.4	77.7** ± **7.5	ns	**0.000** ^‡^	**0.000** ^‡^	**0.008** ^‡^	**0.000** ^**‡**^	**0.000** ^**‡**^
Age at Onset	31.7 ± 10.4	29.2 ± 9	38.6 ± 11.2	N/A	N/A	N/A	N/A	**0.000** ^‡^	N/A	N/A	N/A	N/A
Duration	11.5 ± 7.2	10 ± 6	15.9 ± 8.4	N/A	N/A	N/A	N/A	**0.000** ^‡^	N/A	N/A	N/A	N/A
EDSS	3.5 ± 2.2	2.8 ± 1.9	5.5 ± 1.8	N/A	N/A	N/A	N/A	**0.000** ^‡^	N/A	N/A	N/A	N/A
Number of Relapses	5.1 ± 3.5	5.44 ± 3.7	4.2 ± 3	N/A	N/A	N/A	N/A	**0.049** ^‡^	N/A	N/A	N/A	N/A
Progression Index	0.42 ± 0.54	0.38 ± 0.41	0.53 ± 0.8	N/A	N/A	N/A	N/A	ns	N/A	N/A	N/A	N/A
Anti-Hp positive	60(43.2%)	41(40.2%)	19(51.4%)	33(48.5%)	14(35.9%)	10(47.6%)	ns	ns	ns	ns	ns	ns

Data represent mean ± standard deviation. ^†^p-values were calculated using Pearson Chi-Square or Fisher’s Exact Test (2-sided). ^‡^p-values were calculated using 2-tailed t-test for Equality of Means, equal variances were not assumed. p-values < 0.05 are shown in bold; p-values with a statistical tendency (<0.1) are also shown. Abbreviations: EDSS, expanded disability status scale; F, female; Hp, *Helicobacter pylori*; M, male; ns, not significant; N/A, non-applicable.

### Antibody reactivity to individual Hp antigens

**Figure 1:**
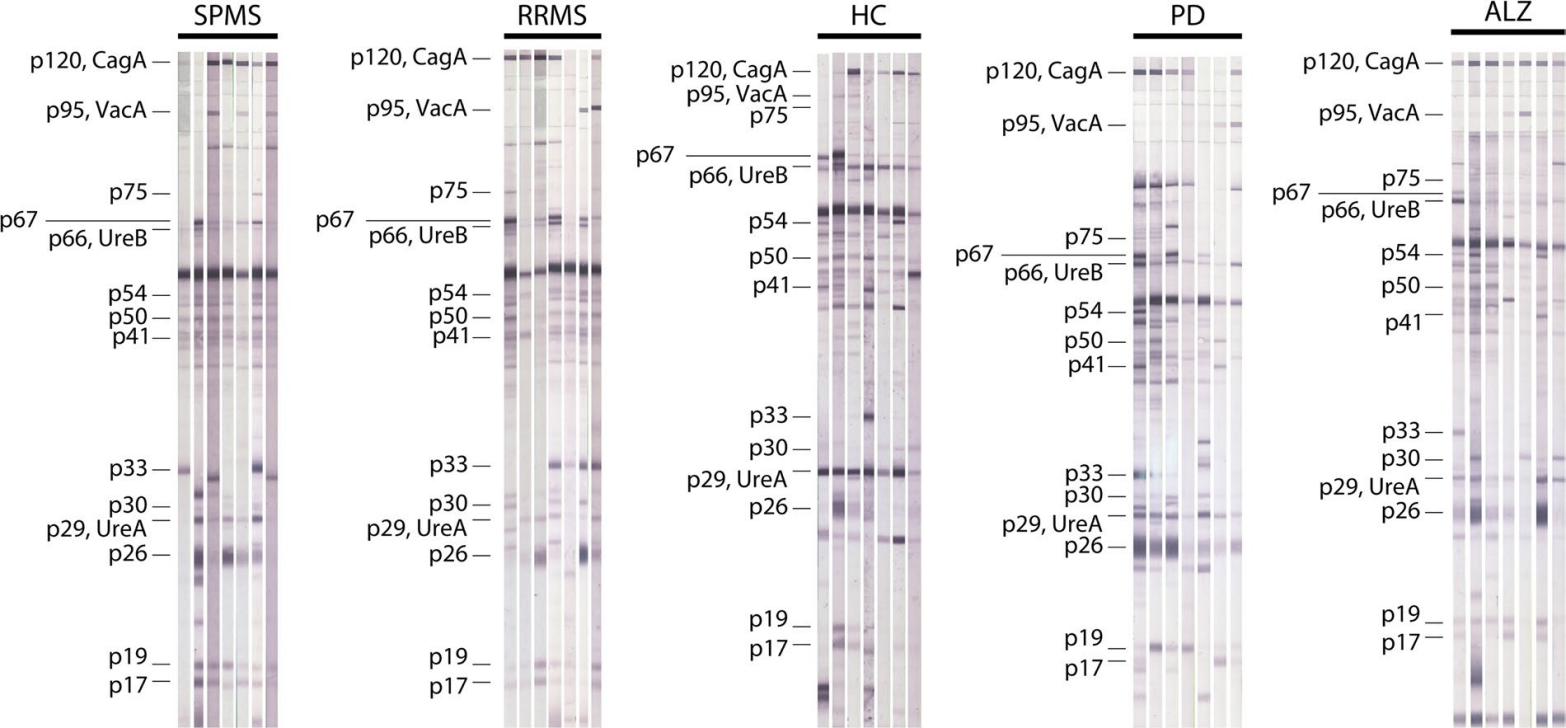
Antibody reactivity against *H*. *pylori* antigens by Western immunoblotting in representative RRMS (n = 7) patients, SPMS (n = 7) patients, healthy controls (HC) (n = 7), patients with Parkinson’s disease (PD) (n = 7) and patients with Alzheimer’s disease (ALZ) (n = 7). Abbreviations: CagA, protein from cytotoxin-associated gene A; UreA, urease A; UreB, urease B; VacA, vacuolating cytotoxin A.

All anti-Hp(+) MS, HCs, PD and ALZ patients were tested for antibody reactivity to individual p120-CagA, p95-VacA (by line immunoassay), p75, p67-FSH, p66-UreB, p54-flagellin, p50, p41, p33, p30-OMP, p29-UreA, p26, p19-OMP, p17 antigens (by Western immunoblot). Figure 1 illustrates results of 35 representative cases (7 RRMS, 7 SPMS, 7 HC, 7 PD and 7 ALZ) and Tables 2, 3 and Supplementary Table 1 summarize results of antibody frequency and magnitude, respectively, in all patients and controls.

**Table 2:** Frequencies of immunoreactive Hp-specific antigens as detected by Western immunoblotting in sera of 60 anti-Hp(+) patients with multiple sclerosis (MS), including 41 relapsing-remitting (RRMS) patients and 19 secondary progressive (SPMS) patients, 33 anti-Hp(+) healthy controls (HC), 14 anti-Hp(+) patients with Parkinson’s disease (PD) and 10 anti-Hp(+) patients with Alzheimer’s disease (ALZ).

Reactive band	All MS (n = 60)	HC (n = 33)	PD (n = 14)	ALZ(n = 10)	RRMS	(n = 41)	SPMS (n = 19)	*p* (All MS vs HC)	*p* (RRMS vs SPMS)	*p* (SPMS vs HC)	*p* (MS vs PD)	*p* (RRMS vs PD)	*p* (SPMS vs PD)	*p* (MS vs ALZ)	*p* (RRMS vs ALZ)	(SPMS vs ALZ)
p120 – CagA	44 (73.3%)	30 (90.9%)	9 (64.3%)	10 (100%)	29 (70.7%)	15 (78.9%)	0.060^†^	ns	**0.042** ^†^	ns	ns	ns	ns	ns	0.092^†^	ns
p95–VacA	17 (28.3%)	4 (12.1%)	5 (35.7%)	3 (30%)	9 (21.9%)	8 (42.1%)	ns	ns	ns	**0.013** ^†^	ns	ns	ns	ns	ns	ns
p75	4 (6.7%)	3 (9.1%)	4 (28.6%)	0	2 (4.9%)	2 (10.5%)	ns	ns	ns	ns	**0.037** ^†^	**0.031** ^†^	ns	ns	ns	ns
p67	33 (55%)	24 (72.7%)	7 (50%)	4 (40%)	19 (46.3%)	14 (73.7%)	ns	ns	**0.022** ^†^	ns	ns	ns	ns	ns	ns	ns
p66–UreB	40 (66.7%)	25 (75.8%)	12 (85.7%)	8 (80%)	26 (63.4%)	14 (73.7%)	ns	ns	ns	ns	ns	ns	ns	ns	ns	ns
p54–flagellin	11 (18.3%)	13 (39.4%)	6 (42.9%)	4 (40%)	4 (9.8%)	7 (36.8%)	**0.026** ^†^	**0.027** ^†^	**0.005** ^†^	ns	0.050^†^	**0.012** ^†^	ns	ns	**0.038** ^†^	ns
p50	32 (53.3%)	19 (57.6%)	10 (71.4%)	6 (60%)	16 (39%)	16 (84.2%)	ns	**0.002** ^†^	ns	0.068^†^	ns	0.061^†^	ns	ns	ns	ns
p41	15 (25%)	16 (48.5%)	10 (71.4%)	6 (60%)	5 (12.2%)	10 (52.6%)	**0.022** ^†^	**0.001** ^†^	**0.001** ^†^	ns	**0.003** ^†^	**0.000** ^†^	ns	0.056^†^	**0.004** ^†^	ns
p33	15 (25%)	9 (27.3%)	4 (28.6%)	1 (10%)	9 (21.9%)	6 (31.6%)	ns	ns	ns	ns	ns	ns	ns	ns	ns	ns
p30	7 (11.7%)	7 (21.2%)	7 (50%)	3 (30%)	4 (9.8%)	3 (15.8%)	ns	ns	ns	ns	**0.001** ^†^	**0.003** ^†^	0.057^†^	ns	ns	ns
p29–UreA	27 (45%)	20 (60.6%)	12 (85.7%)	7 (70%)	13 (31.7%)	14 (73.7%)	ns	**0.002** ^†^	**0.013** ^†^	ns	**0.007** ^†^	**0.001** ^†^	ns	ns	**0.036** ^†^	ns
p26	31 (51.7%)	23 (69.7%)	12 (85.7%)	8 (80%)	20 (48.8%)	11 (57.9%)	ns	ns	0.098^†^	ns	**0.033** ^†^	**0.026** ^†^	ns	ns	0.091^†^	ns
p19	21 (35%)	10 (30.3%)	8 (57.1%)	7 (70%)	15 (36.6%)	6 (31.6%)	ns	ns	ns	ns	ns	ns	ns	0.077^†^	0.079^†^	0.064^†^
p17	23 (38.3%)	10 (30.3%)	5 (35.7%)	4 (40%)	14 (34.1%)	9 (47.4%)	ns	ns	ns	ns	ns	ns	ns	ns	ns	ns

Data represent mean ± standard deviation. ^†^ *p*-values were calculated using Pearson Chi-Square or Fisher’s Exact Test (2-sided). *p*-values < 0.05 are shown in bold; *p*-values with a statistical tendency (<0.1) are also shown. Abbreviations: CagA, protein from cytotoxin-associated gene A; FSH, flagellar sheath protein; ns, not significant; UreA, urease A; UreB, urease B; VacA, vacuolating cytotoxin A.

**Table 3:** Magnitude of antibody responses against immunoreactive Hp-specific antigens as measured by Western immunoblotting in sera of 60 anti-Hp(+) patients with multiple sclerosis (MS), including 41 relapsing-remitting (RRMS) patients and 19 secondary progressive (SPMS) patients, 33 anti-Hp(+) healthy controls (HC), 14 anti-Hp(+) patients with Parkinson’s disease (PD) and 10 anti-Hp(+) patients with Alzheimer’s disease (ALZ).

Reactive band	All MS (n = 60)	RRMS (n = 41)	SPMS (n = 19)	HC (n = 33)	PD (n = 14)	ALZ (n = 10)	*p* (RRMS vs SPMS)	*p* (All MS vs HC)	*p* (RRMS vs HC)	*p* (SPMS vs HC)	*p* (MS vs PD)	*p* (RRMS vs PD)	*p* (SPMS vs PD)	*p* (MS vs ALZ)	*p* (RRMS vs ALZ)	*p* (SPMS vs ALZ)
p120 – CagA	103.8 ± 33.7	103.7 ± 35.2	104.1 ± 31.7	76.6 ± 42	95.7 ± 37.7	66.7 ± 26.4	ns	**0.005** ^‡^	**0.010** ^‡^	**0.019** ^‡^	ns	ns	ns	**0.002** ^‡^	**0.002** ^‡^	**0.004** ^‡^
p95 – VacA	38.1 ± 30.9	38.3 ± 33.4	37.8 ± 30.2	21.3 ± 5.9	32.2 ± 12.9	30.7 ± 16.2	ns	0.051^‡^	ns	ns	ns	ns	ns	ns	ns	ns
p75	36 ± 18.1	35.5 ± 14.8	36.5 ± 27.6	26.3 ± 12.6	24.5 ± 17.9	—	ns	ns	ns	ns	ns	ns	ns	N/A	N/A	N/A
p67	49.2 ± 40	55.1 ± 41.9	41.1 ± 37.2	43.5 ± 34.6	79 ± 40.8	42.3 ± 36.8	ns	ns	ns	ns	ns	ns	**0.036** ^**‡**^	ns	ns	ns
p66 – UreB	45.7 ± 25.3	47.6 ± 27.6	42.2 ± 21.1	65.2 ± 31.7	59 ± 32	45.4 ± 35.6	ns	**0.013** ^‡^	**0.040** ^‡^	**0.010** ^‡^	ns	ns	ns	ns	ns	ns
p54-flagellin	29.8 ± 16.9	27.8 ± 17.4	31 ± 17.9	55.9 ± 39.6	66.8 ± 31.2	42.8 ± 17.6	ns	**0.046** ^‡^	0.067^‡^	0.070^‡^	**0.032** ^‡^	**0.035** ^‡^	**0.039** ^‡^	ns	ns	ns
p50	31.8 ± 16.1	32.9 ± 18.5	30.8 ± 13.8	46.9 ± 22.4	34.5 ± 11.1	33.3 ± 15.7	ns	**0.016** ^‡^	0.051^‡^	**0.014** ^‡^	ns	ns	ns	ns	ns	ns
p41	29.1 ± 15.7	30.6 ± 22	28.4 ± 13	37.5 ± 22.4	41.1 ± 29.3	32 ± 13.5	ns	ns	ns	ns	ns	ns	ns	ns	ns	ns
p33	73.3 ± 35.8	73.9 ± 42.1	72.3 ± 27.6	74.7 ± 34.3	53 ± 42.1	42 ± 0*	ns	ns	ns	ns	ns	ns	ns	N/A	N/A	N/A
p30	35.1 ± 23	22.5 ± 9.8	52 ± 26.3	29.3 ± 13.5	27.9 ± 12.5	37.7 ± 10.1	ns	ns	ns	ns	ns	ns	ns	ns	0.077^‡^	ns
p29 – UreA	36.3 ± 22.4	30.1 ± 20	42 ± 23.7	64.1 ± 49.1	60.8 ± 30.1	34 ± 16.8	ns	**0.026** ^‡^	**0.010** ^‡^	0.092^‡^	**0.022** ^‡^	**0.008** ^‡^	0.096^‡^	ns	ns	ns
p26	56.3 ± 35.5	52.8 ± 34	62.8 ± 38.9	53.5 ± 34	61.6 ± 33.7	46.1 ± 31.7	ns	ns	ns	ns	ns	ns	ns	ns	ns	ns
p19	31.9 ± 14	31.4 ± 14.7	33.2 ± 13.2	42.5 ± 32.8	47.5 ± 24.2	25.6 ± 16.3	ns	ns	ns	ns	ns	ns	ns	ns	ns	ns
p17	34.6 ± 17.3	34.4 ± 17.9	35 ± 17.2	39 ± 21.8	38.8 ± 15.8	34.8 ± 26.3	ns	ns	ns	ns	ns	ns	ns	ns	ns	ns

Data on the magnitude of antibody responses are expressed as mean ± standard deviation arbitratry units (AU) (see methods). ^‡^p-values were calculated using 2-tailed t-test for Equality of Means, equal variances were not assumed. *p*-values < 0.05 are shown in bold; ^*^, n = 1; *p*-values with a statistical tendency (<0.1) are also shown. Abbreviations: CagA, protein from cytotoxin-associated gene A; FSH, Flagellar Sheath Protein; ns, not significant; N/A, non-applicable; UreA, urease A; UreB, urease B; VacA, vacuolating cytotoxin A.

All 14 antigens were recognized at variable frequencies by anti-Hp(+) MS patients and controls (Table 2). The most frequent antibody targets in MS patients were p120-CagA (73.3%), p66-UreB (66.7%), p67-FSH (55%), p50 (53.3%), and p26 (51.7%), being recognized by more than half of MS patients.

Reactivities against p54-flagellin (MS *vs* HC: 18.3 *vs* 39.4%, p = 0.026) and against p41 (MS *vs* HC: 25 *vs* 48.5%, p = 0.022), were less frequent in MS than HCs, and reactivity against CagA exhibited a tendency to lower frequency in MS compared to HCs (73.3 *vs* 90.9%, *p* = 0.06). Reactivities against the remaining 11 of 14 Hp antigens (p95-VacA, p75, p67-FSH, p66-UreB, p50, p33, p30-OMP, p29, p26, p19-OMP, p17) were comparable between MS patients and HC (*p* values corrected for multiple comparisons are given in Supplementary Table 1).

Reactivities against p75 (MS *vs* PD: 6.7 *vs* 28.6%, p = 0.037), p41 (MS *vs* PD: 25 *vs* 71.4%, p = 0.003), p30-OMP (MS *vs* PD: 11.7 *vs* 50%, p = 0.001), p29-UreA (MS *vs* PD: 45 *vs* 85.7%, p = 0.007) and p26 (MS *vs* PD: 51.7 *vs* 85.7%, p = 0.033) were less frequent in MS than PD. Reactivities against the remaining 9 Hp antigens were comparable between MS patients and PD (Table 2 and Supplementary Table 1).

Finally, reactivities against p41 (MS *vs* ALZ: 25 *vs* 60%, p = 0.056) and p19-OMP (MS *vs* ALZ: 35 *vs* 70%, p = 0.077) tended to be less frequent in MS compared to ALZ patients, while reactivities against the remaining 12 Hp antigens were comparable between MS and ALZ patients.

### Anti-Hp reactivity in MS subtypes (RRMS or SPMS)

Anti-Hp antibodies, detected by ELISA, did not differ between RRMS (40.2%) and HCs (48.5%), PD (35.9%) or ALZ (47.6%), or between SPMS (51.4%) and HCs, PD or ALZ (Table 1 and Supplementary Table 1). By blotting, RRMS exhibited greater differences than SPMS patients from HCs. Five of the 14 anti-Hp antigen antibodies were less frequent in RRMS than in HCs (Table 2), including p41 (*p* = 0.001), p54-flagellin (*p* = 0.005), p29-UreA (*p* = 0.013), p67-FSH (*p* = 0.022), and p120-CagA (*p* = 0.042), the latter significance being the only one lost after correcting for multiple comparisons (Supplementary Table 1). Only anti-VacA antibody frequency differed between SPMS and HCs, being higher in SPMS than in HCs (42.1 *vs* 12.1%, *p* = 0.013) (Table 2 and Supplementary Table 1). Anti-VacA antibodies were more frequent is SPMS than in HCs even when only SPMS and HCs over the age of 40 were compared (44.4% SPMS *vs* 13.33% HC *p* = 0.036).

Similarly, RRMS exhibited greater differences than SPMS from PD or ALZ patients. Three of the 14 anti-Hp antigen antibodies were significantly less frequent in RRMS than in both PD and ALZ; p54-flagellin (RRMS *vs* PD: 9.8 *vs* 42.9%, *p* = 0.012), p41 (RRMS *vs* PD: 12.2 *vs* 71.4%, *p* = 0.000; RRMS *vs* ALZ: 12.2 *vs* 60%, *p* = 0.004) and p29-UreA (RRMS *vs* PD: 31.7 *vs* 85.7%, *p* = 0.001; RRMS *vs* ALZ: 31.7 *vs* 70%, *p* = 0.036) (Table 2). These significant differences remained after correction (Supplementary Table 1). Reactivities against 3 additional Hp antigens (p75, p30-OMP and p26) were less frequent (*p* < 0.05 for all) in RRMS than in PD but not ALZ.

### Anti-Hp antibodies in RRMS versus SPMS

The frequency of anti-Hp antibodies by ELISA did not differ between RRMS (40.2%) and SPMS (51.4%) (Table 1). Four of the 14 Hp antigens were recognized to a lesser extent in RRMS patients than in SPMS (Table 2); p54-flagellin (*p* = 0.027), p50 (*p* = 0.002), p41 (*p* = 0.001) and p29-UreA (*p* = 0.002). Also, when comparing RRMS with SPSM patients over the age of 40, we found that these frequencies remained significantly higher in SPMS than RRMS: (p54-flagellin, RRMS: 1/4 (25%), SPMS 7/7 (100%), *p* = 0.019; p50, RRMS: 8/16 (50%), SPMS: 15/16 (93.8%), *p* = 0.017; p41, RRMS: 3/5 (60%), SPMS: 9/10 (90%), *p* = 0.038; p29 = UreA, RRMS: 6/13 (46.2%), SPMS: 13/14 (92.9%), *p* = 0.022).

**Figure 2:**
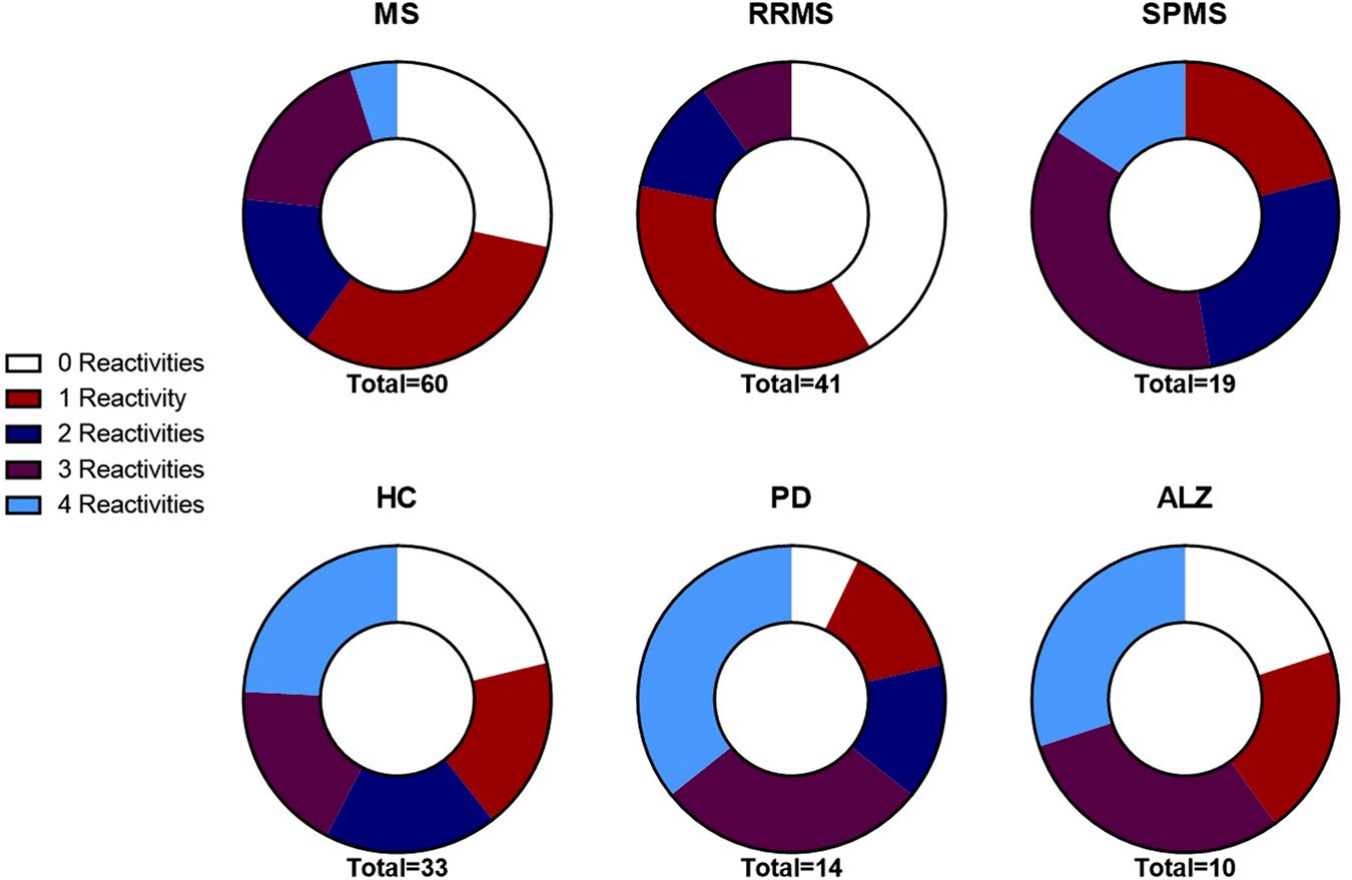
Doughnut charts indicating the proportion of serum samples of total multiple sclerosis (MS), relapse-remitting MS (RRMS), secondary progressive MS (SPMS), healthy controls (HC), Parkinson’s disease (PD) and Alzheimer’s disease (ALZ) patients that were reactive with multiple Hp antigens p54-flagellin, p50, p41 and p29-UreA.

**Figure 3:**
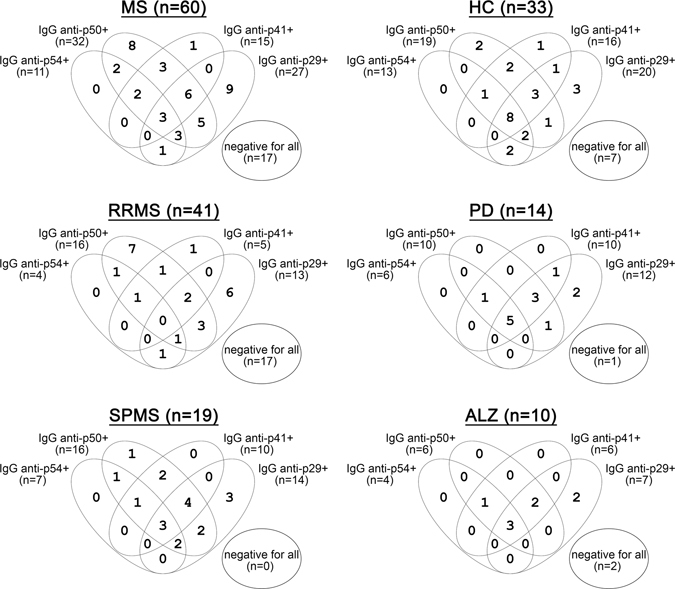
Venn diagrams illustrating the patterns of anti-p54-flagellin, anti-p50, anti-p41 and anti-p29 antibodies overlapping reactivities in patients with MS, RRMS, SPMS, in healthy controls (HC), in patients with Parkinson’s disease (PD) and in patients with Alzheimer’s disease (ALZ).

When comparing the concurrent presence of antibodies against two or more of these four antigens (Fig. 2), we observed that 22% of RRMS patients were reactive to at least two Hp antigens and 78% were reactive to a single antigen or were not reactive (*p* = 0.000) compared to 79% of SPMS patients reactive to at least two Hp antigens and 21% reactive to a single antigen. Overlap of anti-Hp reactivities is shown in Fig. 3.

### Magnitude of anti-Hp response in MS patients and HCs

**Figure 4:**
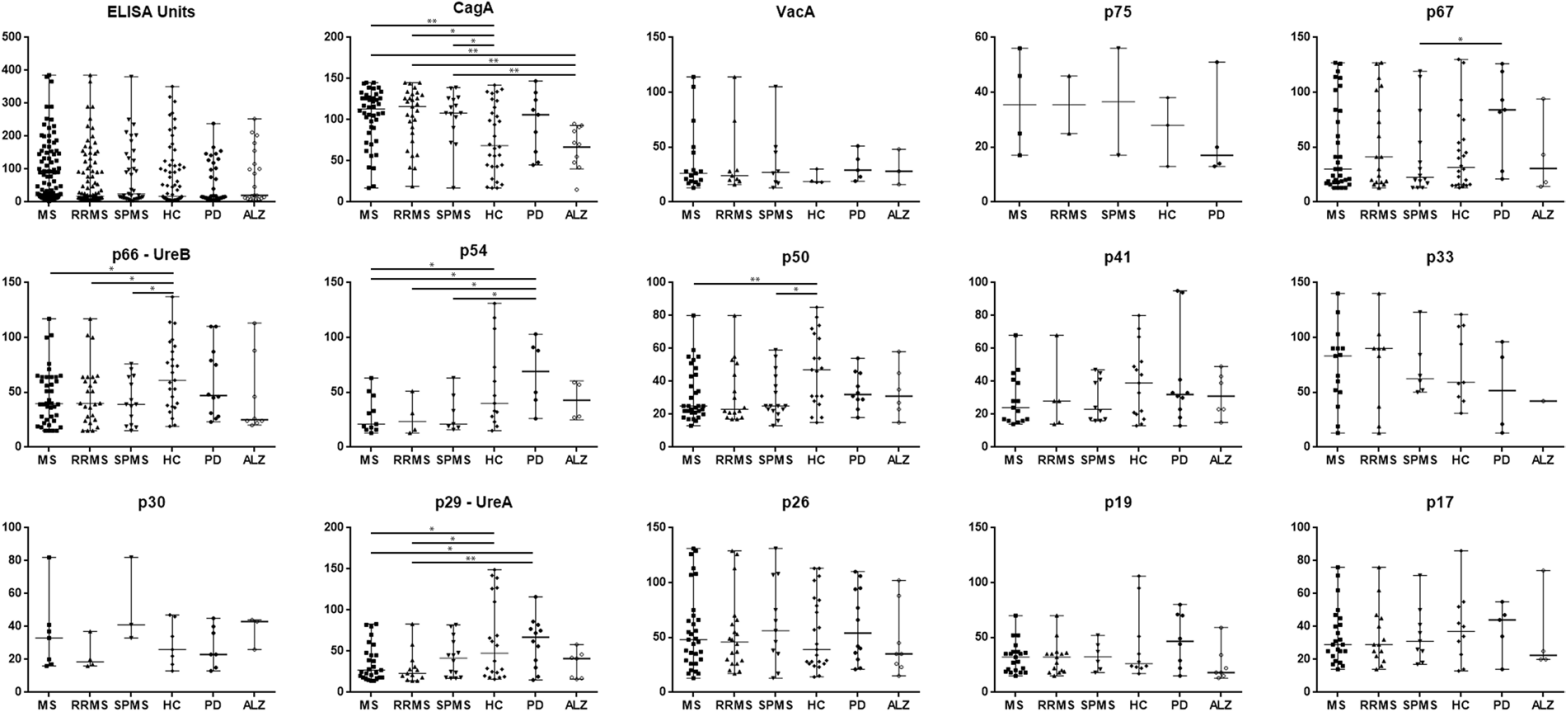
Scatter plots depicting antibody levels against individual Hp antigens in patients with multiple sclerosis (total, RRMS and SPMS), healthy controls (HC), patients with Parkinson’s disease (PD) and patients with Alzheimer’s disease (ALZ). *p < 0.05, **p < 0.01. Abbreviations: CagA, protein from cytotoxin-associated gene A; UreA, urease A; UreB, urease B; VacA, vacuolating cytotoxin A.

Anti-Hp antibody titres by ELISA in individual groups are shown in Fig. 4, whereas levels of reactivity to individual Hp antigens are shown in Table 3. Five anti-Hp reactivities consistently exhibited significant differences between MS or its subtypes and HCs. Stronger responses against p120-CagA were found in MS (RRMS or SPMS) than HCs (*p* < 0.02 for all comparisons, Table 3); less strong responses against p66-UreB were found in MS (RRMS or SPMS) than HCs (*p* < 0.05, for all). When comparing MS *vs* PD or ALZ patients, less strong antibody reactivities were observed against p54-flagellin in MS than in PD and in SPMS than in PD, and against p29-UreA in MS than in PD and in RRMS than in PD, while stronger antibody reactivities were observed against p120-CagA in MS, RRMS and SPMS than in ALZ.

As expected, several correlations were found between anti-Hp antibody levels by ELISA and reactivity to individual Hp antigens, as well as between reactivities to Hp antigens themselves (Supplementary Table 3).

### Clinical correlations of antigen-specific Hp antibody responses

Anti-Hp(+) MS patients (n = 60) were older compared to anti-Hp negative (anti-Hp(−)) MS (n = 79) patients (*p* = 0.007), and age at onset was higher in anti-Hp(+) compared to anti-Hp(−) (p = 0.008) (Supplementary Table 2). Also, female anti-Hp(+) MS patients were older (47.8 ± 12) than female anti-Hp(−) MS patients (41.5 ± 11.6, p = 0.011). In addition, female anti-Hp(+) MS patients had a higher age at onset (35 ± 10.1) compared to female anti-Hp(−) MS (30.3 ± 9.8, p = 0.021). There was no such a difference between male anti-Hp(+) and male anti-Hp(−) MS, or between female anti-Hp(+) and male anti-Hp(+) MS patients.

Several correlations were found between anti-Hp reactivities and clinical features of MS (Table 4). In undivided MS patients, antibodies to p54-flagellin correlated with extended disability status scale (EDSS) (*p* = 0.017), antibodies to p41 correlated with age (*p* = 0.008), antibodies to p33 correlated with age and age at onset (*p* = 0.039 and *p* = 0.024, respectively), antibodies to p29-UreA correlated with EDSS (*p* = 0.005), antibodies to p26 correlated with EDSS and number of relapses (*p* = 0.002 and *p* = 0.037, respectively) and antibodies to p17 correlated with number of relapses (*p* = 0.047). By multiple regression analysis, after adjusting for age, age at onset, duration of disease and sex, statistically significant differences remained for p54-flagellin, p29-UreA and p26 (Supplementary Table 4).

**Table 4:** Clinical correlations of anti-Hp antibody reactivity to individual antigens and features of multiple sclerosis.

		Age at onset	EDSS	Disease duration	PI = EDSS/duration	Relapses	Anti-Hp ELISA Units	CagA	VacA	p75	p67	p66-UreB	p54-flagellin	p50	p41	p33	p30	p29-UreA	p26	p19	p17
Age	R	0.787	0.497	0.530											0.342	0.267					
	p	**0.000**	**0.000**	**0.000**	ns	ns	ns	ns	ns	ns	ns	ns	ns	ns	**0**.**008**	**0.039**	ns	ns	ns	ns	ns
Age at onset	R				0.334	−0.273		.								0.291	0.253				
	p		ns	ns	**0.010**	**0.038**	ns	ns	ns	ns	ns	ns	ns	ns	ns	**0.024**	0.051	ns	ns	ns	ns
EDSS	R			0.522									0.310					0.364	0.392		
	p			**0.000**	ns	ns	ns	ns	ns	ns	ns	ns	**0.017**	ns	ns	ns	ns	**0.005**	**0.002**	ns	ns
Disease duration	R				−0.326	0.243									0.246						
	p				0.012	0.066	ns	ns	ns	ns	ns	ns	ns	ns	0.058	ns	ns	ns	ns	ns	ns
PI	R																				
	p					ns	ns	ns	ns	ns	ns	ns	ns	ns	ns	ns	ns	ns	ns	ns	ns
Relapses	R																	0.242	0.274		0.262
	p						ns	ns	ns	ns	ns	ns	ns	ns	ns	ns	ns	0.067	**0.037**	ns	**0.047**
Anti-Hp ELISA Units	R								0.286	0.312	0.315					0.376	0.375		0.482		0.282
	p							ns	**0.027**	**0.015**	**0.014**	ns	ns	ns	ns	**0.003**	**0.003**	ns	**0.000**	ns	**0.029**

R represents Pearson correlation coefficient. p represents p value (2 tailed) associated with the correlation. *p*-values < 0.05 are shown in bold; *p*-values with a statistical tendency (<0.1) are also shown. Abbreviations: CagA, protein from cytotoxin-associated gene A; EDSS, extended disability status scale; Hp, *Helicobacter* pylori; ns, not significant; PI, Progression Index; UreA, urease A; UreB, urease B; VacA, vacuolating cytotoxin A.

When the analysis was performed in separate RRMS or SPMS patient groups, the following correlations were noted: for RRMS, antibodies to p67 correlated with progression index (PI) (*p* = 0.027), antibodies to p30 correlated with age at onset (*p* = 0.034), antibodies to p30 correlated with PI (*p* = 0.030) and antibodies to p29-UreA correlated with number of relapses (*p* = 0.002). For SPMS, antibodies VacA negatively correlated with age (*p* = 0.031) and antibodies to p66-UreB correlated with EDSS (*p* = 0.046). By multiple regression analysis, after adjusting for age, age at onset, duration of disease and sex, statistically significant differences remained for p29, p26 and p17 in RRMS and for VacA in SPMS (Supplementary Table 4).

Analysis of humoral responses against individual Hp antigens and treatment regimens did not reveal any significant differences (Supplementary Table 5), the only two exceptions being that of RRMS patients treated with natalizumab who had lower anti-p66-UreB antibody responses compared to untreated RRMS patients (27 ± 19.1 *vs* 93.7 ± 27.1, *p* = 0.031) and that of patients on second line treatment who had lower anti-p66-UreB antibody responses compared to untreated RRMS patients (32.9 ± 19.2 *vs* 93.7 ± 27.1, *p* = 0.047).

## Discussion

The present study systematically addressed for the first time the prevalence and clinical significance of anti-Hp reactivity against most immunodominant Hp antigens in consecutive patients with MS, RRMS and SPMS. We found that neither the frequency nor the magnitude of anti-Hp antibody differs between MS patients and HCs or between RRMS and SPMS when ELISA detecting antibodies against the whole Hp extract is used. Previous reports reported conflicting results^1–6, 14^, with most studies reporting lower frequency of anti-Hp antobodies in MS^5, 6, 8–10^, few studies reporting comparable frequencies^7, 14^, and one study reporting higher frequency in MS^1^. Such discepancies may reflect confounding factors, such as ethnicity, sex and age of patients and controls^6, 10^.

However, reactivities against particular Hp immunodominant antigens by immunoblotting revealed several differences between MS and HCs, and more importantly between RRMS and SPMS. First, anti-VacA antibodies were four times more frequent in SPMS than in HCs. This is an intriguing finding as this is the only antigen (amongst 7 with differential recognition between patients and controls) which is recognized more often in MS patients than HCs; all other antigens - including p120-CagA, p67-FSH, p54-flagellin, p50, p41 and p29-UreA- are more frequently recognized in HC than MS group (or its subgroup).

Why VacA, a vacuolating toxin that is excreted by Hp leading to the destruction of epithelial cells, is a frequent target in SPMS is not clear. It could be argued that infection with Hp would initiate antibody (or T cell)-responses against VacA in genetically prone individuals, which in turn could cross-recognize myelin antigens leading to initiation/perpetuation of demyelination in SPMS. Future studies are needed to explore this scenario, including epitope mapping studies of anti-VacA responses in SPMS patients and molecular mimicry studies of cross-reactive VacA and myelin mimics. VacA has been considered a trigger of autoimmunity by molecular mimicry in the past. Goo *et al*. ^18^ have shown that C57BL/6 mice infected with Hp develop high IgG anti-VacA antibodies and autoimmune cholangitis, the murine resemblance of primary biliary cirrhosis. However, we were unable to find any evidence of molecular mimicry and cross-reactivity between VacA and the major PBC autoantigen in patients with PBC^19^. Anti-VacA Hp antibodies have been reported in cerebrospinal fluid (CSF) from patients with Guillain-Barré syndrome, and Miller-Fisher syndrome (MFS), a variant of Guillain-Barré; sequence similarities between VacA and membrane ion transport proteins have been described^20, 21^. Another more plausible explanation for the apparent increase of anti-VacA seroprevalence in SPMS compared to RRMS and HCs is an age-dependent bias. Anti-Hp seroprevalence, in general, as well as that of anti-VacA, increases with age reaching maximum values in individuals around 65 years of age. However, even when we only compared SPMS and HCs over the age of 40, we still found anti-VacA to be more frequent in SPMS than in HCs (44.4% SPMS *vs* 13.33% HC *p* = 0.036), clearly indicating that the over-reactivity against VacA is SPMS-related rather than age-dependent in our cohort. Of relevance, an age-dependent bias may explain why p54-flagellin, p50, p41 and p29-UreA Hp antigens were more frequently recognized in SPMS compared to the younger RRMS patients. Again, when we compared RRMS with SPMS over the age of 40, we have found all these frequencies significantly higher in SPMS than RRMS. The significance of these findings requires further investigation bearing in mind that no difference on the frequency of anti-Hp antibodies against Hp extract containing the totality of antigens was found by ELISA between SPMS and RRMS or between MS and HC. However, we must underline that our study is an association study and does not provide any information regarding the pathogenic potential of anti-Hp antibody responses in MS.

The increased levels of anti-Hp antibodies against CagA in RRMS patients compared to HCs was an unexpected finding. Elevated levels of CagA have been associated with increased risk of gastroduodenal diseases, including ulcer, gastric cancer and MALT lymphoma^22, 23^. Increased levels of anti-CagA Hp antibodies could be attributed to high antigenic burden, as overexpression of CagA correlates with high circulating levels of IL-6 in patients with acute coronary artery disease^24^. Elevated anti-CagA Hp antibodies were detected in immune thrombocytopenic purpura (ITP), whereas reduction of anti-CagA antibodies following eradication therapy in these patients is a prognostic marker of response to ITP treatment and improvement of platelet counts^25^.

Our study revealed several correlations between certain anti-Hp antibody levels and clinical features. The most notable is this between anti-UreA antibody levels and EDSS. Similarly, Pedrini *et al*. found that *H*. *pylori*-infected female MS patients had lower disability scores compared to uninfected female MS patients, but these authors did not assess individual antigens. It is intriguing that levels against 6 of the 14 Hp antigens (p54-flagellin, p41, p33, p29-UreA, p26, p17) correlated with at least one clinical feature of the disease. By multiple regression analysis, statistically significant differences remained for p54-flagellin, p29-UreA and p26.

Three final points must be made. As in other studies, it remains very difficult to sort out the effects of age on the prevalence of anti-microbial antibody reactivities: in our case these are the anti-Hp antibodies in patients with MS. This issue is particularly relevant to the possible association between the increased prevalence of anti-VacA antibodies in SPMS patients noted in the present study. A prospective study evaluating anti-Hp antibodies over time will solve this issue. Second, non-pathogenic factors which may also relate to the enhanced humoral immunity targeting central nervous system antigens may also account for an increased immunity to microbial antigens, suggesting a casual rather a causal effect, but a direct proof of that will be difficult to address. Finally, most of the comparisons in our study pointed at the same basic question in a different way which is the differences in frequencies (or magnitudes) of various Hp antibodies between MS and healthy controls. For this reason, these comparisons may be considered as complementary and some investigators propose that no correction are needed for complementary multiple comparisons. The study by Ridker *et al*. is referenced by statisticians as an example of a study with complementary multiple comparisons not requiring corrections^26^. Nevertheless, even after correction several of the observed differences remained statistically significant (Supplementary revealing that anti-Hp antibody reactivities differ amongst groups and correlate to some extend with clinical parameters). These findings warranty external validation in larger cohorts of patients and controls.

In conclusion, MS patients appear to differentially recognize certain Hp antigens compared to healthy individuals; this recognition differs amongst SPMS and RRMS further underlining the complexity of the potential implication of anti-Hp antibody responses in the immune dysregulation of MS. Larger multi-centre prospective studies are warranted to delineate the puzzling role of *H*. *pylori* in MS, as this could assist clinicians to decide whether patients with MS and evidence of Hp infection need eradication antibiotic treatment or not.

## Methods

### Patients and Controls

Serum samples from 139 consecutive MS patients (98 females, 70.5%; age median 42, range 20-69 years) were tested in the present study, including 102 RRMS (79% of total MS, 70 females, 69%) and 37 SPMS (28 females, 76%). The diagnosis of RRMS was based on the 2010 Revised McDonald criteria^27^. For the transition to the SPMS of the disease we used the criteria introduced by Lorscheider *et al*.^28^. All patients were followed-up at regular intervals in the Out-patient Clinics of the Department of Neurology, University General Hospital of Larissa, University of Thessaly, central Greece between November 2014 and May 2016. Detailed demographic and clinical data are shown in Table 1. Thirty-eight (38) MS patients (27%) were not on any treatment. Amongst the remaining 101 patients (73%), 33 patients (33%) were on interferon-β (including 19 on interferon beta-1α and 14 on interferon beta-1β); 23 on natalizumab (a humanized monoclonal antibody against the cell adhesion molecule α4-integrin); 20 on fingolimod; 18 on glatiramer; 5 on teriflunomide and 2 patients were on mitoxantrone.

Serum samples from healthy controls (HCs) were also tested, consisted of 68 age- and sex-matched individuals (40 females, 58.8%; age median 53.5, range 16–73 years), from a large pool of women and their partners from the Gynecological and Obstetrics Out-patient Clinic of our hospital. HCs had no family history of demyelinating or other autoimmune disorder and no significant comorbidities including gynecological disorders or other chronic illnesses, such as cancer, chronic cardiovascular disease, hypertension, diabetes, or depression.

We also included serum samples from 39 patients with Parkinson’s disease (PD) (20 females, 51.3%; age median 70, range 38–84 years) and 21 patients with Alzheimer’s disease (ALZ) (13 females, 61.9%; age median 77, range 63–89 years), as pathological controls. All patients, healthy controls and pathological controls were white Caucasians.

The investigation conformed to the principles outlined in the Declaration of Helsinki. A written informed consent was obtained from patients and controls. This study was carried out after approval from the Ethical Committee of the University General Hospital of Larissa, Larissa, Greece.

### Anti-Hp antibody testing by ELISA

All MS serum samples were kept in aliquots at −80 °C until used. They were tested for IgG anti-Hp antibodies by ELISA (Euroimmun AG, Lübeck, Germany), as described^14^. Positivity for anti-Hp antibodies was set at >20 relative units (RU)/ml.

### Anti-CagA and anti-VacA antibody testing by line immunoassay

Because CagA and VacA, major virulence factors of Hp infection and immunodominant antigens for anti-Hp antibodies, are insufficiently expressed in the Hp extract (preventing from proper antibody recognition)^14, 29, 30^. recombinant CagA and purified VacA have also been placed as extra lines at the top of the Hp membrane (Euroimmun AG). Anti-CagA and anti-VacA IgG antibody testing was performed by line immunoassay in combination with western blotting for testing of the remaining bands, as described previously (EUROLINE-WB, Euroimmun AG, Lübeck, Germany) ^14, 29, 30^.

### Hp-specific antigen antibody testing by immunoblotting

Reactivity to Hp-specific antigens were tested by western immunoblotting, as previously described^29^ with slight modifications. Pre-testing and titration experiments were performed to allow determination of the proper dilution of serum samples. Blot strips with electrophoretically separated Hp extract (Hp strain ATCC 43504) (Euroimmun AG, Lübeck, Germany) were used as an antigenic source of the Hp proteins. The following 12 antigens were used: p75, p67-FSH, p66-UreB, p54-flagellin, p50, p41, p33, p30-OMP, p29-UreA, p26, p19-OMP, p17. Membranes were incubated with individual serum samples diluted 1/51 for 30 minutes. Following 3 washes for 5 minutes each, the membranes were incubated with pre-determined optimal dilutions of alkaline phosphatase (ALP)-conjugated anti-human IgG antibody (Euroimmun AG). Ready-made NBT/BCIP (Euroimmun AG, Lübeck, Germany) was used as substrate for ALP-conjugated antibodies. Polyclonal antibodies with specificity for Hp antigens (anti-p66-UreB, anti-p29-UreA) were used as representative positive controls to ensure proper antigen-specific antibody testing. The incubated and dried western blot strips were analyzed by means of digital scanning using a flatbed scanner. The reaction area on the membrane is identified by the EUROLineScan software and evaluated to obtain densitometric quantitative data. Absolute grey values are measured by the software and normalized by comparing with the background signal. The intensity (or grey scale level) over the entire western blot strip was defined and the band positions and band intensities were measured, accordingly. Evaluation was made according to the standard reference strip by three independent investigators (GE, CL, EM) who were totally blinded to the clinical data of the patients. The cut-off value for the borderline positives was set at >12 arbitrary units (AU), in accordance to the instruction of the manufacturer.

### Statistical analysis

All data are reported as percentages (%) or median (ranges). Serum levels variation in each patient group was defined by standard deviation (SD). Differences between RRMS patients, SPMS patients and HCs and between groups were tested by two-tailed Student’s *t*-test. Multiple regression analysis was performed after corrections for sex, age, age at onset and duration of the disease. *P*-values smaller than 0.05 were considered significant. All statistical calculations were performed with IBM SPSS Statistics 20 and Graph Pad Prism 5 software.

## Electronic supplementary material

Supplementary information
