# Supplementary material for: Immune responses against Helicobacter pylori-specific antigens differentiate relapsing remitting from secondary progressive multiple sclerosis
Source: Sci Rep. 2017 Aug 11;7:7929. doi: 10.1038/s41598-017-07801-9 (PMC5554191; doi:10.1038/s41598-017-07801-9)
Supplement: Supplementary file 1 — Supplementary information [file 41598_2017_7801_MOESM1_ESM.doc]

**Supplementary Information**

**TITLE:** Immune responses against *Helicobacter pylori*-specific antigens differentiate relapsing remitting from secondary progressive multiple sclerosis

Georgios Efthymiou, Efthymios Dardiotis, Christos Liaskos, Emmanouela Marou, Vana Tsimourtou, Eirini I. Rigopoulou, Thomas Scheper, Alexandros Daponte, Wolfgang Meyer, Lazaros I. Sakkas, Georgios Hadjigeorgiou, Dimitrios P. Bogdanos

**Supplementary Table 1.** Frequencies of immunoreactive Hp-specific antigens as detected by Western immunoblotting in sera of 60 anti-Hp(+) patients with multiple sclerosis (MS), including 41 relapsing-remitting (RRMS) patients and 19 secondary progressive (SPMS) patients, 33 anti-Hp(+) healthy controls (HC), 14 anti-Hp(+) patients with Parkinson’s disease (PD) and 10 anti-Hp(+) patients with Alzheimer’s disease (ALZ).

| Reactive band | All MS  (n=60) | HC  (n=33) | PD  (n=14) | ALZ  (n=10) | RRMS  (n=41) | SPMS  (n=19) | *p*  (All ms vs HC) | *p*  (rrms vs spms) | *p*  (RRMS vs HC) | *p*  (SPMS vs HC) | *p*  (MS vs PD) | *p*  (RRMS vs PD) | *p*  (SPMS vs PD) | *p*  (MS vs ALZ) | *p*  (RRMS vs ALZ) | *p*  (SPMS vs ALZ) |
| --- | --- | --- | --- | --- | --- | --- | --- | --- | --- | --- | --- | --- | --- | --- | --- | --- |
| p120 – CagA | 44 (73.3%) | 30 (90.9%) | 9 (64.3%) | 10 (100%) | 29 (70.7%) | 15 (78.9%) | 0.060^†^ | ns | **0.042**^†^ | ns | ns | ns | ns | ns | 0.092^†^ | ns |
| Yate’s Continuity Correction* | | | | | | | 0.081 |  | 0.064 |  |  |  |  |  | 0.123 |  |
| p95 – VacA | 17 (28.3%) | 4 (12.1%) | 5 (35.7%) | 3 (30%) | 9 (21.9%) | 8 (42.1%) | ns | ns | ns | **0.013**^†^ | ns | ns | ns | ns | ns | ns |
| Yate’s Continuity Correction | | | | | | |  |  |  | **0.033** |  |  |  |  |  |  |
| p75 | 4 (6.7%) | 3 (9.1%) | 4 (28.6%) | 0 | 2 (4.9%) | 2 (10.5%) | ns | ns | ns | ns | **0.037**^†^ | **0.031**^†^ | ns | ns | ns | ns |
| Yate’s Continuity Correction | | | | | | |  |  |  |  | 0.058 | **0.050** |  |  |  |  |
| p67 | 33 (55%) | 24 (72.7%) | 7 (50%) | 4 (40%) | 19 (46.3%) | 14 (73.7%) | ns | ns | **0.022**^†^ | ns | ns | ns | ns | ns | ns | ns |
| Yate’s Continuity Correction | | | | | | |  |  | **0.040** |  |  |  |  |  |  |  |
| p66 – UreB | 40 (66.7%) | 25 (75.8%) | 12 (85.7%) | 8 (80%) | 26 (63.4%) | 14 (73.7%) | ns | ns | ns | ns | ns | ns | ns | ns | ns | ns |
| Yate’s Continuity Correction | | | | | | |  |  |  |  |  |  |  |  |  |  |
| p54 | 11 (18.3%) | 13 (39.4%) | 6 (42.9%) | 4 (40%) | 4 (9.8%) | 7 (36.8%) | **0.026**^†^ | **0.027**^†^ | **0.005**^†^ | ns | **0.050**^†^ | **0.012**^†^ | ns | ns | **0.038**^†^ | ns |
| Yate’s Continuity Correction | | | | | | | **0.048** | **0.030** | **0.006** |  | 0.107 | **0.018** |  |  | 0.061 |  |
| p50 | 32 (53.3%) | 19 (57.6%) | 10 (71.4%) | 6 (60%) | 16 (39%) | 16 (84.2%) | ns | **0.002**^†^ | ns | 0.068^†^ | ns | 0.061^†^ | ns | ns | ns | ns |
| Yate’s Continuity Correction | | | | | | |  | **0.003** |  | 0.096 |  | 0.074 |  |  |  |  |
| p41 | 15 (25%) | 16 (48.5%) | 10 (71.4%) | 6 (60%) | 5 (12.2%) | 10 (52.6%) | **0.022**^†^ | **0.001**^†^ | **0.001**^†^ | ns | **0.003**^†^ | **0.000**^†^ | ns | 0.056^†^ | **0.004**^†^ | ns |
| Yate’s Continuity Correction | | | | | | | **0.039** | **0.002** | **0.001** |  | **0.003** | **0.000** |  | 0.062 | **0.004** |  |
| p33 | 15 (25%) | 9 (27.3%) | 4 (28.6%) | 1 (10%) | 9 (21.9%) | 6 (31.6%) | ns | ns | ns | ns | ns | ns | ns | ns | ns | ns |
| Yate’s Continuity Correction | | | | | | |  |  |  |  |  |  |  |  |  |  |
| p30 | 7 (11.7%) | 7 (21.2%) | 7 (50%) | 3 (30%) | 4 (9.8%) | 3 (15.8%) | ns | ns | ns | ns | **0.001**^†^ | **0.003**^†^ | 0.057^†^ | ns | ns | ns |
| Yate’s Continuity Correction | | | | | | |  |  |  |  | **0.004** | **0.004** | 0.084 |  |  |  |
| p29 – UreA | 27 (45%) | 20 (60.6%) | 12 (85.7%) | 7 (70%) | 13 (31.7%) | 14 (73.7%) | ns | **0.002**^†^ | **0.013**^†^ | ns | **0.007**^†^ | **0.001**^†^ | ns | ns | **0.036**^†^ | ns |
| Yate’s Continuity Correction | | | | | | |  | **0.006** | **0.024** |  | **0.014** | **0.001** |  |  | **0.063** |  |
| p26 | 31 (51.7%) | 23 (69.7%) | 12 (85.7%) | 8 (80%) | 20 (48.8%) | 11 (57.9%) | ns | ns | 0.098^†^ | ns | **0.033**^†^ | **0.026**^†^ | ns | ns | 0.091^†^ | ns |
| Yate’s Continuity Correction | | | | | | |  |  |  |  | **0.043** | **0.035** |  |  | 0.154 |  |
| p19 | 21 (35%) | 10 (30.3%) | 8 (57.1%) | 7 (70%) | 15 (36.6%) | 6 (31.6%) | ns | ns | ns | ns | ns | ns | ns | 0.077^†^ | 0.079^†^ | 0.064^†^ |
| Yate’s Continuity Correction | | | | | | |  |  |  |  |  |  |  | 0.081 | 0.119 | 0.113 |
| p17 | 23 (38.3%) | 10 (30.3%) | 5 (35.7%) | 4 (40%) | 14 (34.1%) | 9 (47.4%) | ns | ns | ns | ns | ns | ns | ns | ns | ns | ns |
| Yate’s Continuity Correction | | | | | | |  |  |  |  |  |  |  |  |  |  |

Data represent mean ± standard deviation. ^†^*p*-values were calculated using Pearson Chi-Square or Fisher’s Exact Test (2-sided). *p*-values <0.05 are shown in bold; *p*-values with a statistical tendency (<0.1) are also shown. *Yate’s Continuity Correction values are also included for statistically significant differences arising from Pearson Chi-Square tests. Abbreviations: CagA, protein from cytotoxin-associated gene A; FSH, flagellar sheath protein; ns, not significant; UreA, urease A; UreB, urease B; VacA, vacuolating cytotoxin A.

**Supplementary Table 2.** Major demographic and clinical characteristics of 139 patients with multiple sclerosis (MS), including 102 with relapsing remitting MS (RRMS) and 37 with secondary progressive MS (SPMS). Patients have been stratified in accordance to their anti-*Helicobacter pylori* (Hp) antibody seroprevalence tested by ELISA.

| Characteristics | Total  (n=139) | Hp(+)  (n=60) | Hp(-)  (n=79) | *p*  Hp(+) *vs* Hp(-) | HC  (n=68) | *p*  MS *vs* HC |
| --- | --- | --- | --- | --- | --- | --- |
| Sex (M/F) | 41(29.5%)/  98(70.5%) | 18 (30%)/42 (70%) | 23 (29.1%)/56 (70.9%) | ns | 28(41.2%)/  40(58.8%) | ns |
| Age | 43.2 ± 12 | 46.4 ± 12.3 | 40.8 ± 11.2 | **0.007**^‡^ | 47.4 ± 16.9 | 0.071^‡^ |
| Age on Onset | 31.7 ± 10.4 | 34.4 ± 10.5 | 29.7 ± 10 | **0.008**^‡^ |  |  |
| (>20/≤20 years) | 138 (99.3%)/1 (0.7%) | 60 (100%)/0 | 78 (98.7%)/1 (1.3%) | ns | 58 (85.3%)/10 (14.7%) | **0.000**^†^ |
| (>30/≤30 years) | 116 (83.5%)/23 (16.5%) | 54 (90%)/6 (10%) | 62 (78.5%)/17 (21.5%) | ns | 57 (83.8%)/11 (16.2%) | ns |
| (>40/≤40 years) | 77 (55.4%)/62 (44.6%) | 37 (61.7%)/23 (38.3%) | 40 (50.6%)/39 (49.4%) | ns | 53 (77.9%)/15 (22.1%) | **0.002**^†^ |
| (>50/≤50 years) | 42 (30.2%)/97 (69.8%) | 24 (40%)/36 (60%) | 18 (22.8%)/61 (77.2%) | **0.040**^†^ | 44 (64.7%)/24 (35.3%) | **0.000**^†^ |
| MS Type  RRMS (yes, %)  SPMS (yes, %) | 102 (73.4%)  37 (26.6%) | 41 (68.3%)  19 (31.7%) | 61 (77.2%)  18 (22.8%) | ns |  |  |
| Duration of disease | 11.5 ± 7.2 | 12 ± 7.6 | 11.1 ± 6.9 | ns |
| EDSS | 3.5 ± 2.2 | 3.6 ± 2.3 | 3.5 ± 2.2 | ns |
| Number of Relapses | 5.1 ± 3.5 | 4.5 ± 3.7 | 5.6 ± 3.3 | ns |
| Progression Index | 0.42 ± 0.54 | 0.44 ± 0.79 | 0.41 ± 0.39 | ns |
| Anti-Hp ELISA (RU/ml) | 65.6 ± 85.5 | 136.3 ± 90 | 11.7 ± 4.8 | **0.000**^‡^ | 71.2 ± 91.2 | ns |
| Anti-Hp positive | 60(43.2%) |  |  |  | 33(48.5%) | ns |

Data represent mean ± standard deviation. ^†^*p*-values were calculated using Fisher’s Exact Test (2-sided). ^‡^*p*-values were calculated using 2-tailed t-test for Equality of Means, equal variances are not assumed. *p*-values <0.05 are shown in bold; *p*-values with a statistical tendency (<0.1) are also shown. Abbreviations: M, male; F, female; EDSS, expanded disability status scale; RRMS, relapsing-remitting multiple sclerosis; SPMS, secondary progressive multiple sclerosis.

**Supplementary Table 3.** Correlations of antibody levels between individual Hp antigens

|  | | ELISA Units | CagA | VacA | p75 | p67 | p66-UreB | p54 | p50 | p41 | p33 | p30 | p29-UreA | p26 | p19 | p17 |
| --- | --- | --- | --- | --- | --- | --- | --- | --- | --- | --- | --- | --- | --- | --- | --- | --- |
| ELISA Units | R |  |  | .286 | .312 | .315 |  |  |  |  | .376 | .375 |  | .482 |  | .282 |
|  | p |  | ns | **.027** | **.015** | **.014** | ns | ns | ns | ns | **.003** | **.003** | ns | **.000** | ns | **.029** |
| CagA | R |  |  |  | .252 | .321 |  |  |  |  | -.042 |  |  |  |  |  |
|  | p |  |  | ns | **.**052 | **.012** | ns | ns | ns | ns | ns | ns | ns | ns | ns | ns |
| VacA | R |  |  |  | .498 |  |  |  |  |  | .247 |  |  | .303 |  | .258 |
|  | p |  |  |  | **.000** | ns | ns | ns | ns | ns | .057 | ns | ns | **.019** | ns | **.046** |
| p75 | R |  |  |  |  |  |  |  |  |  |  |  |  | .293 |  | .522 |
|  | p |  |  |  |  | ns | ns | ns | ns | ns | ns | ns | ns | **.023** | ns | **.000** |
| p67 | R |  |  |  |  |  | .354 |  |  | .267 | .416 |  |  | .248 |  | .370 |
|  | p |  |  |  |  |  | **.006** | ns | **.000** | **.039** | **.001** | **.000** | ns | .056 | ns | **.004** |
| p66-UreB | R |  |  |  |  |  |  |  | .249 | .306 |  | .313 |  |  |  |  |
|  | p |  |  |  |  |  |  | ns | .055 | **.018** | ns | **.015** | ns | ns | ns | ns |
| p54 | R |  |  |  |  |  |  |  |  |  |  |  | .295 |  |  |  |
|  | p |  |  |  |  |  |  |  | ns | ns | ns | ns | **.022** | ns | ns | ns |
| p50 | R |  |  |  |  |  |  |  |  | .613 | .456 | .343 | .302 |  |  |  |
|  | p |  |  |  |  |  |  |  |  | **.000** | **.000** | **.007** | **.019** | ns | ns | ns |
| p41 | R |  |  |  |  |  |  |  |  |  | .330 | .345 | .345 |  |  |  |
|  | p |  |  |  |  |  |  |  |  |  | **.010** | **.007** | **.007** | ns | ns | ns |
| p33 | R |  |  |  |  |  |  |  |  |  |  | .288 |  |  |  |  |
|  | p |  |  |  |  |  |  |  |  |  |  | **.026** | ns | ns | ns | ns |
| p30 | R |  |  |  |  |  |  |  |  |  |  |  | .332 | .342 | .328 | .390 |
|  | p |  |  |  |  |  |  |  |  |  |  |  | **.010** | **.008** | **.011** | **.002** |
| p29-UreA | R |  |  |  |  |  |  |  |  |  |  |  |  | .476 | .353 | .451 |
|  | p |  |  |  |  |  |  |  |  |  |  |  |  | **.000** | **.006** | **.000** |
| p26 | R |  |  |  |  |  |  |  |  |  |  |  |  |  | .336 | .483 |
|  | p |  |  |  |  |  |  |  |  |  |  |  |  |  | **.009** | **.000** |
| p19 | R |  |  |  |  |  |  |  |  |  |  |  |  |  |  | .539 |
|  | p |  |  |  |  |  |  |  |  |  |  |  |  |  |  | **.000** |
| p17 | R |  |  |  |  |  |  |  |  |  |  |  |  |  |  |  |
|  | p |  |  |  |  |  |  |  |  |  |  |  |  |  |  |  |

R represents Pearson correlation coefficient. p represents p value (2 tailed) associated with the correlation. *p*-values <0.05 are shown in bold; *p*-values with a statistical tendency (<0.1) are also shown. Abbreviations: CagA, protein from cytotoxin-associated gene A; EDSS, extended disability status scale; ns, not significant; UreA, urease A; UreB, urease B; VacA, vacuolating cytotoxin A.

**Supplementary Table 4.** Clinical correlations of anti-Hp antibody reactivity to individual antigens and features of multiple sclerosis followed by regression analysis after adjusting for age, age at onset, disease duration and sex.

| **MS** | **RRMS** | **SPMS** |
| --- | --- | --- |
|  |  | VacA-age (R=-0.496, *p*=**0.031**)  adj. for age (R=N/A, *p*=N/A)  adj. age at onset (R=-0.543, p=**0.20**)  adj. for disease duration (R=-0.476, p=**0.046**)  adj. for sex (R=-0.513, p=**0.029**) |
|  | p67-PI (R=0.345, *p*=**0.027**)  adj. for age (R=0.352, p=**0.026**)  adj. age at onset ((R=0.320, p=**0.044**)  adj. for disease duration (R=0.311, p=0.051)  adj. for sex (R=0.341, p=**0.031**) |  |
|  |  | p66-UreB-EDSS (R=0.477, *p*=**0.046**)  adj. for age (R=0.493, p=**0.044**)  adj. age at onset (R=0.488, p=**0.047**)  adj. for disease duration (R=0.573, p=**0.016**)  adj. for sex (R=0.481, p=0.051) |
| p54-EDSS (R=0.310, *p*=**0.017**)  adj. for age (R=0.309, *p*=**0.018**)  adj. age at onset (R=0.316, *p*=**0.016**)  adj. for disease duration (R=0.279, *p*=**0.034**)  adj. for sex (R=0.312, *p*=**0.017**) |  |  |
| p41-age (R=0.342, *p*=**0.008**)  adj. for age (R=N/A, *p*=N/A)  adj. age at onset (R=0.273, *p*=**0.036**)  adj. for disease duration (R=0.257, *p*=0.050)  adj. for sex (R=0.357, *p*=**0.005**) |  |  |
| p33-age (R=0.267, *p*=**0.039**)  adj. for age (R=N/A, *p*=N/A)  adj. age at onset (R=0.065, *p*=0.622)  adj. for disease duration (R=0.294, *p*=**0.024**)  adj. for sex (R=0.266, *p*=**0.033**) |  |  |
| p33-age at onset (R=0.291, *p*=**0.024**)  adj. for age (R=0.135, *p*=0.307)  adj. age at onset (R=N/A, *p*=N/A)  adj. for disease duration (R=0.297, *p*=**0.023**)  adj. for sex (R=0.289, *p*=**0.026**) |  |  |
|  | p30-PI (R=0.340, *p*=**0.030**)  adj. for age (R=0.362, p=**0.022**)  adj. age at onset (R=0.301, p=0.059)  adj. for disease duration (R=0.314, p=**0.049**)  adj. for sex (R=0.338, p=**0.033**) |  |
|  | p30-age at onset (R=0.331, *p*=**0.034**)  adj. for age (R=0.285, p=0.074)  adj. age at onset (R=N/A, *p*=N/A)  adj. for disease duration (R=0.316, p=**0.047**)  adj. for sex (R=0.326, p=**0.040**) |  |
| p29-UreA-EDSS (R=0.364, *p*=**0.005**)  adj. for age (R=0.327, *p*=**0.012**)  adj. age at onset (R=0.364, *p*=**0.006**)  adj. for disease duration (R=0.316, *p*=**0.016**)  adj. for sex (R=0.262, *p*=**0.045**) |  |  |
|  | p29-UreA-relapses (R=0.471, *p*=**0.002**)  adj. for age (R=0.468, p=**0.002**)  adj. age at onset (R=0.459, p=**0.003**)  adj. for disease duration (R=0.472, p=**0.002**)  adj. for sex (R=0.450, p=**0.004**) |  |
| p26-EDSS (R=0.392, *p*=**0.002**)  adj. for age (R=0.393, *p*=**0.002**)  adj. age at onset (R=0.397, *p*=**0.002**)  adj. for disease duration (R=0.316, *p*=**0.016**)  adj. for sex (R=0.397, *p*=**0.002**) | p26-EDSS (R=0.437, *p*=**0.004**)  adj. for age (R=0.423, p=**0.006**)  adj. age at onset (R=0.437, p=**0.005**)  adj. for disease duration (R=0.416, p=**0.008**)  adj. for sex (R=0.439, p=**0.005**) |  |
| p26-relapses (R=0.274, *p*=**0.037**)  adj. for age (R=0.288, *p*=**0.030**)  adj. age at onset (R=0.291, *p*=**0.028**)  adj. for disease duration (R=0.242, *p*=0.069)  adj. for sex (R=0.250, *p*=0.061) | p26-relapses (R=0.371, *p*=**0.017**)  adj. for age (R=0.385, p=**0.014**)  adj. age at onset (R=0.396, p=**0.011**)  adj. for disease duration (R=0.346, p=**0.029**)  adj. for sex (R=0.346, p=**0.029**) |  |
| p17-relapses (R=0.262, *p*=**0.047**)  adj. for age (R=0.273, *p*=**0.040**)  adj. age at onset (R=0.300, *p*=**0.023**)  adj. for disease duration (R=0.261, *p*=0.050)  adj. for sex (R=0.238, *p*=0.075) | p17-relapses (R=0.345, *p*=**0.027**)  adj. for age (R=0.349, p=**0.027**)  adj. age at onset (R=0.326, p=**0.024**)  adj. for disease duration (R=0.339, p=**0.032**)  adj. for sex (R=0.318, p=**0.046**) |  |

R represents Pearson correlation coefficient. p represents p value (2 tailed) associated with the correlation. *p*-values <0.05 are shown in bold; *p*-values with a statistical tendency (<0.1) are also shown. Abbreviations: adj., adjusted; EDSS, extended disability status scale; Hp, *Helicobacter* pylori; N/A, not applicable; PI, Progression Index; UreA, urease A; UreB, urease B; VacA, vacuolating cytotoxin A.

**Supplementary Table 5.** Analysis of antibody reactivities (frequency and magnitude expressed as arbitrary units (AU)) in relation to type of treatment. Data represent percentages or mean ± standard deviation. ^*^p-values were calculated using Pearson Chi-Square or Fisher’s Exact Test (2-sided). **p-values were calculated using 2-tailed t-test for Equality of Means, equal variances were not assumed. p-values <0.05 are shown in bold; p-values with a statistical tendency (<0.1) are also shown. Abbreviations: CagA, protein from cytotoxin-associated gene A; Hp, *Helicobacter pylori*; ns, not significant; RRMS, relapse-remitting multiple sclerosis; SPMS, secondary progressive multiple sclerosis; UreA, urease A; UreB, urease B; VacA, vacuolating cytotoxin A.

| **RRMS** |  | **Hp(+) (n=41)** | **Hp(-) (n=61)** | **p** |
| --- | --- | --- | --- | --- |
|  | **Treated (n=89)** | 36 (87.7%) | 53 (86.9%) | ns |
|  | **Untreated (n=13)** | 5 (12.2%) | 8 (13.1%) |  |

| **RRMS** |  | **anti-Hp ELISA units** | **p** |
| --- | --- | --- | --- |
|  | **Treated (n=89)** | 56 ± 76.3 | ns |
|  | **Untreated (n=13)** | 86.5 ± 116.9 |  |

| **Hp(+) RRMS** |  | **anti-Hp ELISA units** | **p** |
| --- | --- | --- | --- |
|  | **Treated (n=36)** | 120.8 ± 85.8 | ns |
|  | **Untreated (n=5)** | 208 ±105 |  |

| **Hp(+) RRMS** | **Positivity for individual bands** | **Untreated (n=5)** | **Treated (n=36)** | **p** |
| --- | --- | --- | --- | --- |
|  | **p120 - CagA** | 3 | 26 | ns |
|  | **p95 - VacA** | 1 | 8 | ns |
|  | **p75** | 0 | 2 | ns |
|  | **p67** | 3 | 16 | ns |
|  | **p66 - UreB** | 3 | 23 | ns |
|  | **p54** | 0 | 4 | ns |
|  | **p50** | 2 | 14 | ns |
|  | **p41** | 1 | 4 | ns |
|  | **p33** | 3 (60%) | 6 (16.7%) | 0.061* |
|  | **p30** | 1 | 3 | ns |
|  | **p29 - UreA** | 3 | 10 | ns |
|  | **p26** | 2 | 18 | ns |
|  | **p19** | 2 | 13 | ns |
|  | **p17** | 2 | 12 | ns |

| **Hp(+) RRMS** | **Magnitude of individual bands** | **Untreated (n=5)** | **Treated (n=36)** | **p** |
| --- | --- | --- | --- | --- |
|  | **p120 - CagA** | 99 ± 38.4 | 104.2 ± 35.6 | ns |
|  | **p95 - VacA** | 20 | 40.6 ± 34.9 | ns |
|  | **p75** | 0 | 35.5 ± 14.8 | - |
|  | **p67** | 46 ± 52 | 56.8 ± 41.6 | ns |
|  | **p66 - UreB** | 93.7 ± 27.1 | 41.6 ± 21.7 | ns |
|  | **p54** | 0 | 27.8 ± 17.4 | - |
|  | **p50** | 34.5 ± 23.3 | 32.7 ± 18.8 | ns |
|  | **p41** | 68 | 21.3 ± 7.8 | ns |
|  | **p33** | 76.7 ± 35 | 72.5 ± 48.3 | ns |
|  | **p30** | 20 | 23.3 ± 11.8 | ns |
|  | **p29 - UreA** | 41.3 ± 36.1 | 26.7 ± 13.7 | ns |
|  | **p26** | 32 ± 17 | 55.1 ± 35 | ns |
|  | **p19** | 27.5 ± 13.4 | 32 ± 15.3 | ns |
|  | **p17** | 38 ± 12.7 | 33.8 ± 19 | ns |

| **RRMS** |  | **Hp(+) (n=41)** | **Hp(-) (n=61)** | **p _Untreated_ *_vs_* _Natalizumab_** | **p _Untreated_ *_vs_* _Glatiramer_** | **p _Untreated_ *_vs_* _Interferons_** | **p _Untreated_ *_vs_* _Fingolimod_** | **p Untreated *vs* Mitoxantrone/Teriflunomide** |
| --- | --- | --- | --- | --- | --- | --- | --- | --- |
|  | **Untreated (n=13)** | 5 (12.2%) | 8 (13.1%) | ns | ns | ns | ns | ns |
|  | **Natalizumab (n=23)** | 9 (22%) | 14 (23%) |  |  |  |  |  |
|  | **Glatiramer (n=15)** | 5 (12.2%) | 10 (16.4%) |  |  |  |  |  |
|  | **Interferons (n=28)** | 11 (26.8%) | 17 (27.9%) |  |  |  |  |  |
|  | **Fingolimod (n=18)** | 9 (22%) | 9 (14.8%) |  |  |  |  |  |
|  | **Mitoxantrone/Teriflunomide (n=5)** | 2 (4/9%) | 3 (4.9%) |  |  |  |  |  |

| **RRMS** |  | **anti-Hp ELISA units** | **p _Untreated_ *_vs_* _Natalizumab_** | **p _Untreated_ *_vs_* _Glatiramer_** | **p _Untreated_ *_vs_* _Interferons_** | **p _Untreated_ *_vs_* _Fingolimod_** | **p Untreated *vs* Mitoxantrone/Teriflunomide** |
| --- | --- | --- | --- | --- | --- | --- | --- |
|  | **Untreated (n=13)** | 86.5 ± 116.9 | ns | ns | ns | ns | ns |
|  | **Natalizumab (n=23)** | 39.6 ± 46.6 |  |  |  |  |  |
|  | **Glatiramer (n=15)** | 41.6 ± 53.4 |  |  |  |  |  |
|  | **Interferons (n=28)** | 64.8 ± 85.8 |  |  |  |  |  |
|  | **Fingolimod (n=18)** | 79.9 ± 106.2 |  |  |  |  |  |
|  | **Mitoxantrone/Teriflunomide (n=5)** | 38.8 ± 52.7 |  |  |  |  |  |

| **Hp(+) RRMS** |  | **anti-Hp ELISA units** | **p _Untreated_ *_vs_* _Natalizumab_** | **p _Untreated_ *_vs_* _Glatiramer_** | **p _Untreated_ *_vs_* _Interferons_** | **p _Untreated_ *_vs_* _Fingolimod_** | **p Untreated *vs* Mitoxantrone/Teriflunomide** |
| --- | --- | --- | --- | --- | --- | --- | --- |
|  | **Untreated (n=5)** | 208 ± 105 | 0.054** | 0.093** | ns | ns | ns |
|  | **Natalizumab (n=9)** | 83.4 ± 48.9 |  |  |  |  |  |
|  | **Glatiramer (n=5)** | 102.4 ± 55 |  |  |  |  |  |
|  | **Interferons (n=11)** | 143.9 ± 92.3 |  |  |  |  |  |
|  | **Fingolimod (n=9)** | 149.5 ± 114.3 |  |  |  |  |  |
|  | **Mitoxantrone/Teriflunomide (n=)** | 79.1 ± 75.4 |  |  |  |  |  |

| **Hp(+) RRMS** | **Positivity for individual bands** | **Untreated (n=5)** | **Natalizumab (n=9)** | **Glatiramer**  **(n=5)** | **Interferons**  **(n=11)** | **Fingolimod**  **(n=9)** | **Mitoxantrone/Teriflunomide**  **(n=2)** | **p _Untreated_ *_vs_* _Natalizumab_** | **p _Untreated_ *_vs_* _Glatiramer_** | **p _Untreated_ *_vs_* _Interferons_** | **p _Untreated_ *_vs_* _Fingolimod_** | **p Untreated *vs* Mitoxantrone/Teriflunomide** |
| --- | --- | --- | --- | --- | --- | --- | --- | --- | --- | --- | --- | --- |
|  | **p120 - CagA** | 3 | 7 | 4 | 8 | 5 | 2 | ns | ns | ns | ns | ns |
|  | **p95 - VacA** | 1 | 1 | 2 | 3 | 1 | 1 | ns | ns | ns | ns | ns |
|  | **p75** | 0 | 0 | 1 | 1 | 0 | 0 | - | ns | ns | - | - |
|  | **p67** | 3 (60%) | 4 (44.4%) | 4 (80%) | 6 (54.5%) | 1 (11.1%) | 1 (50%) | ns | ns | ns | 0.095* | ns |
|  | **p66 - UreB** | 3 | 6 | 4 | 6 | 6 | 1 | ns | ns | ns | ns | ns |
|  | **p54** | 0 | 0 | 1 | 1 | 2 | 0 | ns | ns | ns | ns | - |
|  | **p50** | 2 | 1 | 2 | 8 | 2 | 1 | ns | ns | ns | ns | ns |
|  | **p41** | 1 | 1 | 1 | 1 | 0 | 1 | ns | ns | ns | ns | ns |
|  | **p33** | 3 (60%) | 2 (22.2%) | 0 | 2 (19.2%) | 1 (11.1%) | 1 (50%) | ns | ns | ns | 0.095* | ns |
|  | **p30** | 1 | 0 | 1 | 2 | 0 | 0 | ns | ns | ns | ns | - |
|  | **p29 - UreA** | 3 | 2 | 1 | 3 | 3 | 1 | ns | ns | ns | ns | ns |
|  | **p26** | 2 | 5 | 2 | 4 | 6 | 1 | ns | ns | ns | ns | ns |
|  | **p19** | 2 | 3 | 1 | 4 | 3 | 2 | ns | ns | ns | ns | ns |
|  | **p17** | 2 | 4 | 1 | 3 | 3 | 1 | ns | ns | ns | ns | ns |

| **Hp(+) RRMS** | **Magnitude of individual bands** | **Untreated (n=5)** | **Natalizumab (n=9)** | **Glatiramer**  **(n=5)** | **Interferons**  **(n=11)** | **Fingolimod**  **(n=9)** | **Mitoxantrone/Teriflunomide**  **(n=2)** | **p _Untreated_ *_vs_* _Natalizumab_** | **p _Untreated_ *_vs_* _Glatiramer_** | **p _Untreated_ *_vs_* _Interferons_** | **p _Untreated_ *_vs_* _Fingolimod_** | **p Untreated *vs* Mitoxantrone/Teriflunomide** |
| --- | --- | --- | --- | --- | --- | --- | --- | --- | --- | --- | --- | --- |
|  | **p120 - CagA** | 99 ± 38.4 | 96.4 ± 44.7 | 109 ± 36 | 106 ± 35.1 | 123.4 ± 14.4 | 66.5 ± 34.6 | ns | ns | ns | ns | ns |
|  | **p95 - VacA** | 20 | 19 | 18.5 ± 3.5 | 43.7 ± 26.3 | 24 | 114 | - | - | - | - | - |
|  | **p75** | 0 | 0 | 46 | 25 | 0 | 0 | - | - | - | - | - |
|  | **p67** | 46 ± 52 | 36.3 ± 18.2 | 66.8 ± 48.4 | 74.8 ± 48.2 | 18 | 30 | ns | ns | ns | - | - |
|  | **p66 - UreB** | 93.7 ± 27.1 | 27 ± 19.1 | 47.3 ± 17.5 | 55.5 ± 25.2 | 38.8 ± 18.9 | 39 | **0.031**** | 0.075** | ns | 0.051** | - |
|  | **p54** | 0 | 0 | 16 | 13 | 41 ± 14 | 0 | - | - | - | - | - |
|  | **p50** | 34.5 ± 23.3 | 44 | 36.5 ± 26.2 | 33.5 ± 21.9 | 19.5 ± 3.5 | 34 | - | ns | ns | ns | - |
|  | **p41** | 68 | 14 | 28 | 28 | 0 | 15 | - | - | - | - | - |
|  | **p33** | 76.7 ± 35 | 54.5 ± 50.2 | 0 | 115 ± 35.4 | 13 | 83 | ns |  | ns | - | - |
|  | **p30** | 20 | 0 | 16 | 27 ± 14.1 | 0 | 0 | - | - | - | - | - |
|  | **p29 - UreA** | 41.3 ± 36.1 | 20.5 ± 3.5 | 58 | 18.7 ± 7.2 | 28 ± 12.8 | 28 | ns | - | ns | ns | - |
|  | **p26** | 32 ± 17 | 45.4 ± 22.2 | 57 ± 12.7 | 82.5 ± 43.7 | 47.2 ± 41.7 | 37 | ns | ns | ns | ns | - |
|  | **p19** | 27.5 ± 13.4 | 35 ± 17 | 36 | 22.8 ± 8.7 | 44 ± 22.9 | 26 ± 8.5 | ns | - | ns | ns | ns |
|  | **p17** | 38 ± 12.7 | 36.5 ± 7.3 | 76 | 34.3 ± 24.4 | 18.3 ± 4 | 25 | ns | - | ns | ns | - |

| **RRMS** | **Treatment** | **Hp+ (n=41)** | **Hp- (n=61)** | **p _Untreated_ *_vs_* _First_** | **p _Untreated_ *_vs_* _Second_** | **p _Untreated_ *_vs_* _Interferons_** |
| --- | --- | --- | --- | --- | --- | --- |
|  | **Untreated (n=13)** | 5 (12.2%) | 8 (13.1%) | ns | ns | ns |
|  | **First line (n=20)** | 7 (17.1%) | 13 (21,3%) |  |  |  |
|  | **Second line (n=41)** | 18 (43.9%) | 23 (37.7%) |  |  |  |
|  | **Interferons (n=28)** | 11 (26.8%) | 17 (27.9%) |  |  |  |

| **RRMS** |  | **anti-Hp ELISA units** | **p _Untreated_ *_vs_* _First_** | **p _Untreated_ *_vs_* _Second_** | **p _Untreated_ *_vs_* _Interferons_** |
| --- | --- | --- | --- | --- | --- |
|  | **Untreated (n=13)** | 86.5 ± 116.9 | ns | ns | ns |
|  | **First line (n=20)** | 40.9 ± 51.9 |  |  |  |
|  | **Second line (n=41)** | 57.3 ± 80 |  |  |  |
|  | **Interferons (n=28)** | 64.8 ± 85.8 |  |  |  |

| **Hp(+) RRMS** |  | **anti-Hp ELISA units** | **p _Untreated_ *_vs_* _First_** | **p _Untreated_ *_vs_* _Second_** | **p _Untreated_ *_vs_* _Interferons_** |
| --- | --- | --- | --- | --- | --- |
|  | **Untreated (n=5)** | 208 ± 105 | 0.075** | ns | ns |
|  | **First line (n=7)** | 95.8 ± 55.6 |  |  |  |
|  | **Second line (n=18)** | 116.4 ± 91.8 |  |  |  |
|  | **Interferons (n=)** | 143.9 ± 92.3 |  |  |  |

| **Hp(+) RRMS** | **Positivity for individual bands** | **Untreated (n=5)** | **First line (n=7)** | **Second line (n=18)** | **Interferons (n=11)** | **p _Untreated_ *_vs_* _First_** | **p _Untreated_ *_vs_* _Second_** | **p _Untreated_ *_vs_* _Interferons_** |
| --- | --- | --- | --- | --- | --- | --- | --- | --- |
|  | **p120 - CagA** | 3 | 6 | 12 | 8 | ns | ns | ns |
|  | **p95 - VacA** | 1 | 3 | 2 | 3 | ns | ns | ns |
|  | **p75** | 0 | 1 | 0 | 1 | ns | - | ns |
|  | **p67** | 3 | 5 | 5 | 6 | ns | ns | ns |
|  | **p66 - UreB** | 3 | 5 | 12 | 6 | ns | ns | ns |
|  | **p54** | 0 | 1 | 2 | 1 | ns | ns | ns |
|  | **p50** | 2 | 3 | 3 | 8 | ns | ns | ns |
|  | **p41** | 1 | 2 | 1 | 1 | ns | ns | ns |
|  | **p33** | 3 (60%) | 1 (14.3%) | 3 (16.7%) | 2 (18.2%) | ns | 0.089* | ns |
|  | **p30** | 1 | 1 | 0 | 2 | ns | ns | ns |
|  | **p29 - UreA** | 3 | 2 | 5 | 3 | ns | ns | ns |
|  | **p26** | 2 | 3 | 11 | 4 | ns | ns | ns |
|  | **p19** | 2 | 3 | 6 | 4 | ns | ns | ns |
|  | **p17** | 2 | 2 | 7 | 3 | ns | ns | ns |

| **Hp(+) RRMS** | **Magnitude of individual bands** | **Untreated (n=5)** | **First line (n=7)** | **Second line (n=18)** | **Interferons (n=11)** | **p _Untreated_ *_vs_* _First_** | **p _Untreated_ *_vs_* _Second_** | **p _Untreated_ *_vs_* _Interferons_** |
| --- | --- | --- | --- | --- | --- | --- | --- | --- |
|  | **p120 - CagA** | 99 ± 38.4 | 94.8 ± 38.7 | 107.7 ± 36.8 | 106 ± 35.1 | ns | ns | ns |
|  | **p95 - VacA** | 20 | 21.5 ± 3.5 | 50.3 ± 55.2 | 43.7 ± 26.3 | - | - | - |
|  | **p75** | 0 | 46 | 0 | 25 | - | - | - |
|  | **p67** | 46 ± 52 | 59.4 ± 45.1 | 32.6 ± 17.8 | 74.8 ± 48.2 | ns | ns | ns |
|  | **p66 - UreB** | 93.7 ± 27.1 | 45.6 ± 15.6 | 32.9 ± 19.2 | 55.5 ± 25.2 | 0.072** | **0.047**** | ns |
|  | **p54** | 0 | 16 | 41 ± 14.1 | 13 | - | - | - |
|  | **p50** | 34.5 ± 23.3 | 35.7 ± 18.6 | 27.7 ± 14.4 | 33.5 ± 21.9 | ns | ns | ns |
|  | **p41** | 68 | 21.5 ± 9.2 | 14 | 28 | - | - | - |
|  | **p33** | 76.7 ± 35 | 83 | 40.7 ± 42.8 | 115 ± 35.4 | - | ns | ns |
|  | **p30** | 20 | 16 | 0 | 27 ± 14.1 | - | - | - |
|  | **p29 - UreA** | 41.3 ± 36.1 | 43 ± 21.2 | 25 ± 10.1 | 18.7 ± 7.2 | ns | ns | ns |
|  | **p26** | 32 ± 17 | 50.3 ± 14.6 | 46.4 ± 32.7 | 82.5 ± 43.7 | ns | ns | ns |
|  | **p19** | 27.5 ± 13.4 | 29.3 ± 8.3 | 39.5 ± 18.7 | 22.8 ± 8.7 | ns | ns | ns |
|  | **p17** | 38 ± 12.7 | 50.5 ± 36.1 | 28.7 ± 11.3 | 34.3 ± 24.4 | ns | ns | ns |

| **SPMS** |  | **Hp+ (n=19)** | **Hp- (n=18)** | **p** |
| --- | --- | --- | --- | --- |
|  | **Treated (n=12)** | 8 (42.1%) | 4 (22.2%) | ns |
|  | **Untreated (n=25)** | 11 (57.9%) | 14 (77.8%) |  |

| **SPMS** |  | **anti-Hp ELISA units** | **p** |
| --- | --- | --- | --- |
|  | **Treated (n=12)** | 105.4 ± 91.7 | ns |
|  | **Untreated (n=25)** | 69.4 ± 92.8 |  |

| **Hp(+) SPMS** |  | **anti-Hp ELISA units** | **p** |
| --- | --- | --- | --- |
|  | **Treated (n=8)** | 153 ± 73.8 | ns |
|  | **Untreated (n=11)** | 142.4 ± 100.7 |  |

| **Hp(+) SPMS** | **Positivity for individual bands** | **Untreated (n=11)** | **Treated (n=8)** | **p** |
| --- | --- | --- | --- | --- |
|  | **p120 - CagA** | 8 | 7 | ns |
|  | **p95 - VacA** | 4 | 4 | ns |
|  | **p75** | 2 | 0 | ns |
|  | **p67** | 8 | 6 | ns |
|  | **p66 - UreB** | 9 | 5 | ns |
|  | **p54** | 5 | 2 | ns |
|  | **p50** | 10 | 6 | ns |
|  | **p41** | 6 | 4 | ns |
|  | **p33** | 2 | 4 | ns |
|  | **p30** | 1 | 2 | ns |
|  | **p29 - UreA** | 7 | 7 | ns |
|  | **p26** | 6 | 5 | ns |
|  | **p19** | 3 | 3 | ns |
|  | **p17** | 5 | 4 | ns |

| **Hp(+) SPMS** | **Magnitude of individual bands** | **Untreated (n=11)** | **Treated (n=8)** | **p** |
| --- | --- | --- | --- | --- |
|  | **p120 - CagA** | 102.3 ± 40 | 106.1 ± 21.8 | ns |
|  | **p95 - VacA** | 47.8 ± 40.3 | 27.8 ± 15.4 | ns |
|  | **p75** | 36.5 ± 27.6 | - | - |
|  | **p67** | 36.3 ± 36 | 47.7 ± 41.3 | ns |
|  | **p66 - UreB** | 43.1 ± 24.4 | 40.6 ± 15.8 | ns |
|  | **p54** | 21.4 ± 6.7 | 55 ± 11.3 | ns |
|  | **p50** | 29.1 ± 12.9 | 33.5 ± 16 | ns |
|  | **p41** | 25.2 ± 11.7 | 33.3 ± 15 | ns |
|  | **p33** | 58.5 ± 9.2 | 79.3 ± 32.5 | ns |
|  | **p30** | 82 | 37 ± 5.7 | 0.097*** |
|  | **p29 - UreA** | 42.9 ± 23 | 41.1 ± 26.1 | ns |
|  | **p26** | 79.3 ± 44.4 | 43 ± 20.2 | ns |
|  | **p19** | 38.7 ± 15.9 | 27.7 ± 9.5 | ns |
|  | **p17** | 36.4 ± 23.5 | 33.3 ± 6.8 | ns |
